# Radioiodine labeling and in vivo trafficking of extracellular vesicles

**DOI:** 10.1038/s41598-021-84636-5

**Published:** 2021-03-03

**Authors:** Chae Moon Hong, Prakash Gangadaran, Ji Min Oh, Ramya Lakshmi Rajendran, Arunnehru Gopal, Liya Zhu, Byeong-Cheol Ahn

**Affiliations:** 1 Department of Nuclear Medicine, School of Medicine, Kyungpook National University, 130 Dongdeok-ro, Jung Gu, Daegu, 41944 Republic of Korea; 2 Department of Nuclear Medicine, Kyungpook National University Hospital, Daegu, Republic of Korea; 3 BK21 FOUR KNU Convergence Educational Program of Biomedical Sciences for Creative Future Talents, School of Medicine, Kyungpook National University, Daegu, Republic of Korea

**Keywords:** Biotechnology, Nanoscience and technology

## Abstract

Biodistribution and role of extracellular vesicles (EVs) are still largely unknown. Reliable tracking methods for EVs are needed. In this study, nuclear imaging using radioiodine were developed and applied for tracking EVs derived from cell lines. EVs were obtained from supernatant of thyroid cancer cell (Cal62) and natural killer cells (NK92-MI) using sequential ultracentrifuges. Sulfosuccinimidyl-3-(4-hydroxypheynyl) propionate were labeled to membrane of Cal62 and NK92-MI cell derived EVs, then the EVs were labeled with radioiodine (I-131 and I-125) using pre-coated iodination tubes (RI-EVs). In vivo gamma camera images were obtained after intravenous injection of the RI-EVs, and ex vivo biodistribution study was also performed. EVs were labeled with radioiodine and radiochemical purity of the RI-EV was more than 98%. Results of nanoparticle tracking analysis and electron microscopy showed that there was no significant difference in EVs before and after the radioiodine labeling. After intravenous injection of RI-EVs to mice, gamma camera imaging well visualized the real-time biodistribution of the RI-EVs. RI-EVs were mainly visualized at liver, spleen, and lung. Nuclear imaging system of EVs derived from thyroid cancer and NK cells using radioiodine labeling of the EVs was established. Thus, this system might be helpful for in vivo tracking of EVs.

## Introduction

Extracellular vesicles (EVs) are naturally released from the cell that are delimited by a lipid bilayer and cannot replicate, which do not contain functional nucleus^1^. EVs are found in bodily fluids such as blood, urine, plasma, and they are released from tumor cells, immune cells and almost every cell. EVs have been called as diverse terms, such as exosome, ectosome, microvesicle, outer membrane vesicle, apoptotic bodies and etc. EVs are containing different kinds of proteins, lipids, nucleic acids, and other cell components^2^. Now a days, EVs are considered as a mediator of cellular communication and delivering their cargos to distant cells^3, 4^.

As EVs are released from various diseases including cancers, they can be used as diagnostic biomarkers of the diseases^3^. Recent studies also showed that possibility of EVs as therapeutic armors and their biological functions are quiet variable depends on parent cells of EVs. Immune cell derived EVs demonstrated cytotoxic effect to various cancers, and stem cell derived EVs induced accelerated regenerations of various organs^5–7^. For the development of EV therapeutic strategies, in vivo trafficking of the EV in living subjects is essential. Despite intensive researches about EVs, only a few studies analyzed EV biodistribution in vivo animal models^2^. Therefore, development of appropriate EV imaging strategies in live animal is urgently needed for successful clinical translation of therapeutics of EVs.

Bioluminescence and fluorescence imaging techniques are widely used for in vivo trafficking EVs^8–10^. However, these optical imaging techniques have a disadvantage of limited tissue penetration power, that there are difficulties in tracking EVs in deep internal organs^11, 12^. To overcome the limitation, nuclear imaging can be an alternative option for in vivo EV trafficking. Radionuclide labeled cells or other biomaterials are successfully in vivo monitored by using gamma camera, single photon emission computed tomography (SPECT) or positron emission tomography (PET)^11^. Gamma rays emitted from these radionuclides have higher penetration power than optical signals, that radionuclide imaging is more suitable to visualize EVs. In addition, the imaging technologies using radionuclide provide accurate quantitative measurements even in vivo study^2^. Even though, nuclear imaging system also have some limitations (relatively low spatial resolution, hazard of the using ionizing radiation, and higher cost), nuclear imaging modalities are already widely used in clinics, so that, human application of nuclear imaging for in vivo EV trafficking is much easier than other imaging modalities due to less hurtles of safety and regulation issues. In addition, the images can be combined with computed tomography (CT) or magnetic resonance imaging (MRI) for accurate anatomical localization. Even though the nuclear imaging has been widely used for in vivo tracking of cells and other biomaterials^13–15^, only several reports were published for tracking EVs using the nuclear imaging^16–19^.

In this study, the labeling method of radioiodine to EVs was established, and characterizations of EVs before and after the radioiodine labeling were performed. In vivo imaging of the radiolabeled EVs using gamma camera was also performed in normal mice, and corresponding results were compared between in vivo imaging and biodistribution study.

## Results

### Radioiodine labeling of EVs

**Figure 1:**
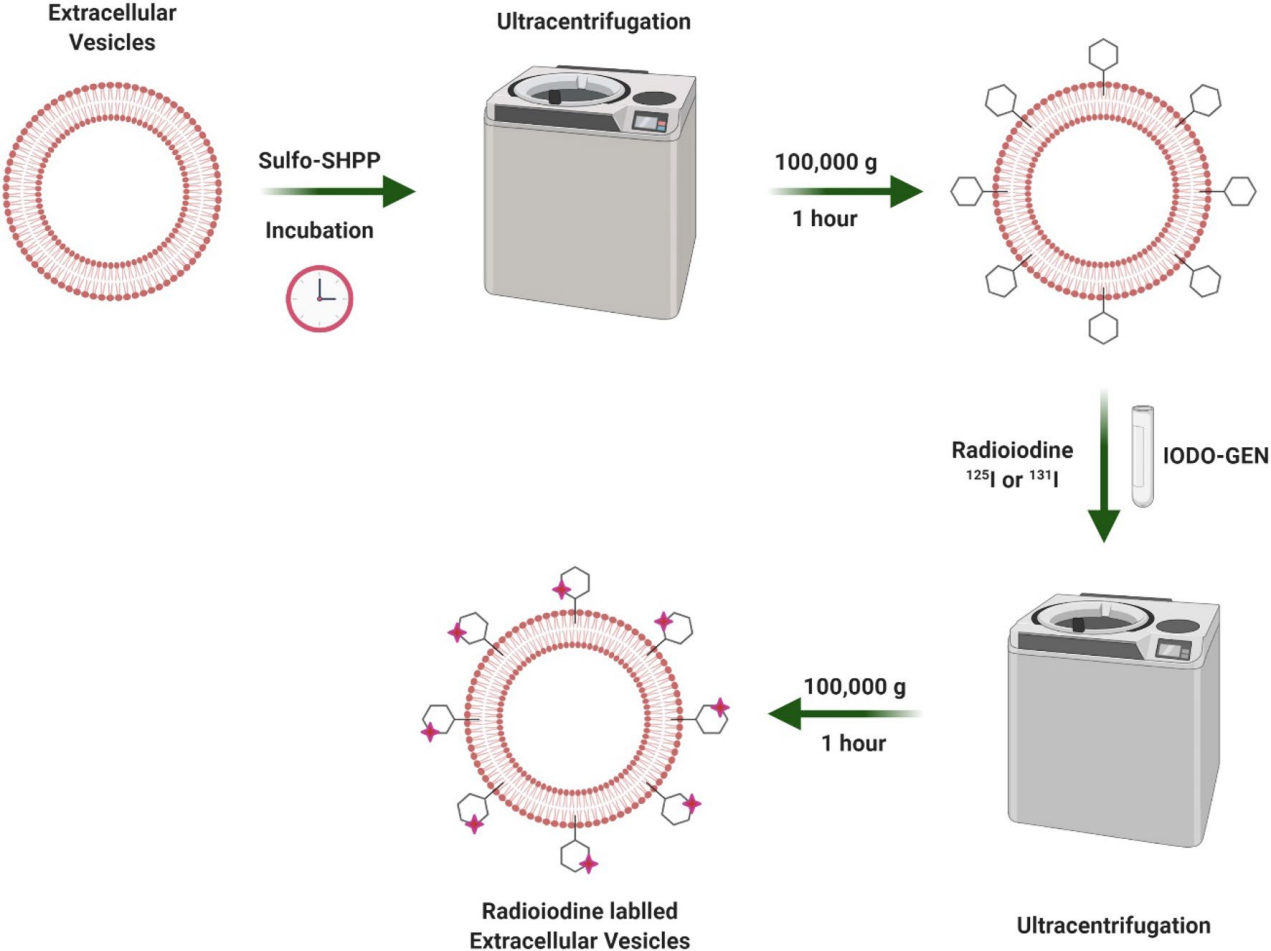
Schematic diagram of radioiodine labeling of extracellular vesicles. Created with BioRender.com.

**Figure 2:**
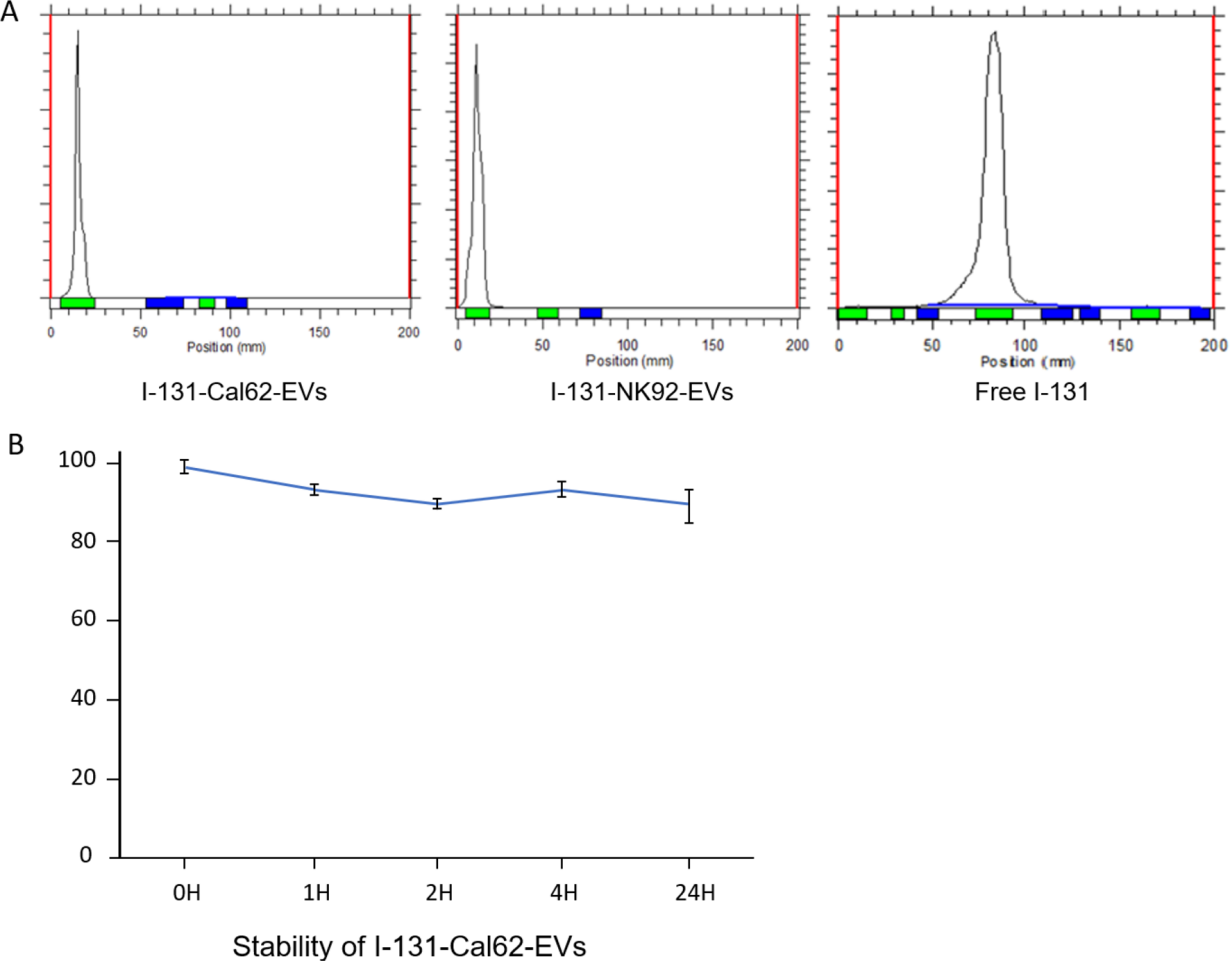
Radiochemical purity and stability of radioiodine labeled EVs. (**A**) Instant thin layer chromatography (ITLC) showed good radiochemical purity after radioiodine labeling. Radiochemical purity of I-131-Cal62-EVs and I-131-NK-EVs after purification were 98.9 ± 1.8% (n = 3) and 98.6 ± 0.9% (n = 3). (**B**) Stability of I-131-Cal62-EVs was examined in 20% fetal bovine serum (n = 3). The level of stability was analyzed by ITLC. Data are presented as mean ± standard deviation.

After purification of EVs, I-131 was successfully labeled to EVs (Fig. 1). Radiochemical yield of I-131 labeled Cal62 derived EVs (I-131-Cal62-EVs) and I-131 labeled NK92-MI derived EVs (I-131-NK92-EV) was 27.7 ± 6.8% and 30.2 ± 7.7%. Radiochemical purity of I-131-Cal62-EVs and I-131-NK92-EVs after purification were 98.9 ± 1.8% and 98.6 ± 0.9% (Fig. 2A). The stability of I-131-EVs in serum was tested (Fig. 2B). The percentage of I-131-Cal62-EVs in 20% FBS was 92.9 ± 1.1% (1 h), 89.9 ± 0.8% (2 h), 93.5 ± 1.7% (4 h) and 89.0 ± 4.5% (24 h), respectively.

### Characterizations of EVs after radioiodine labeling

**Figure 3:**
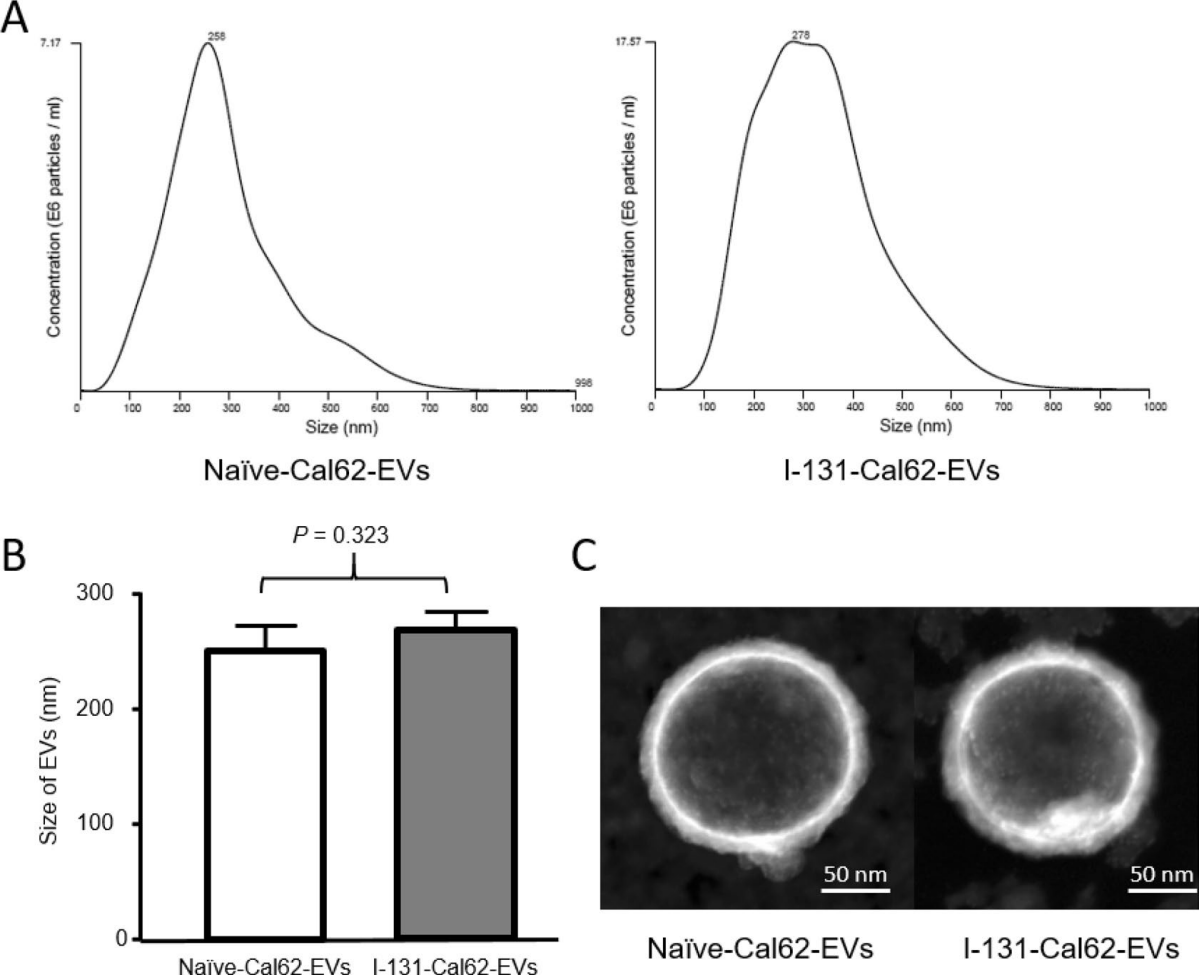
Characterization of I-131-Cal62-EVs. (**A**) Size distribution of naïve-Cal62-EVs and I-131-Cal62-EVs were examined by nanoparticle tracking analysis. (**B**) There was no significant difference of the average sizes between naïve-Cal62-EVs and I-131-Cal62-EVs. (**C**) Scanning electron microscopy images revealed that there were no significant differences of morphology and size between naïve-Cal62-EVs and I-131-Cal62-EVs. Data are presented as mean ± standard deviation.

There were no significant differences in the sizes between naïve-Cal62-EVs (255.7 ± 18.6 nm) and I-131-Cal62-EVs (272.0 ± 16.8 nm), measured by nanoparticle tracking analysis (NTA) (Fig. 3A,B). Morphology and size of Cal62-EVs were also not significantly different by Scanning electron microscopy (SEM) images (Fig. 3C).

**Figure 4:**
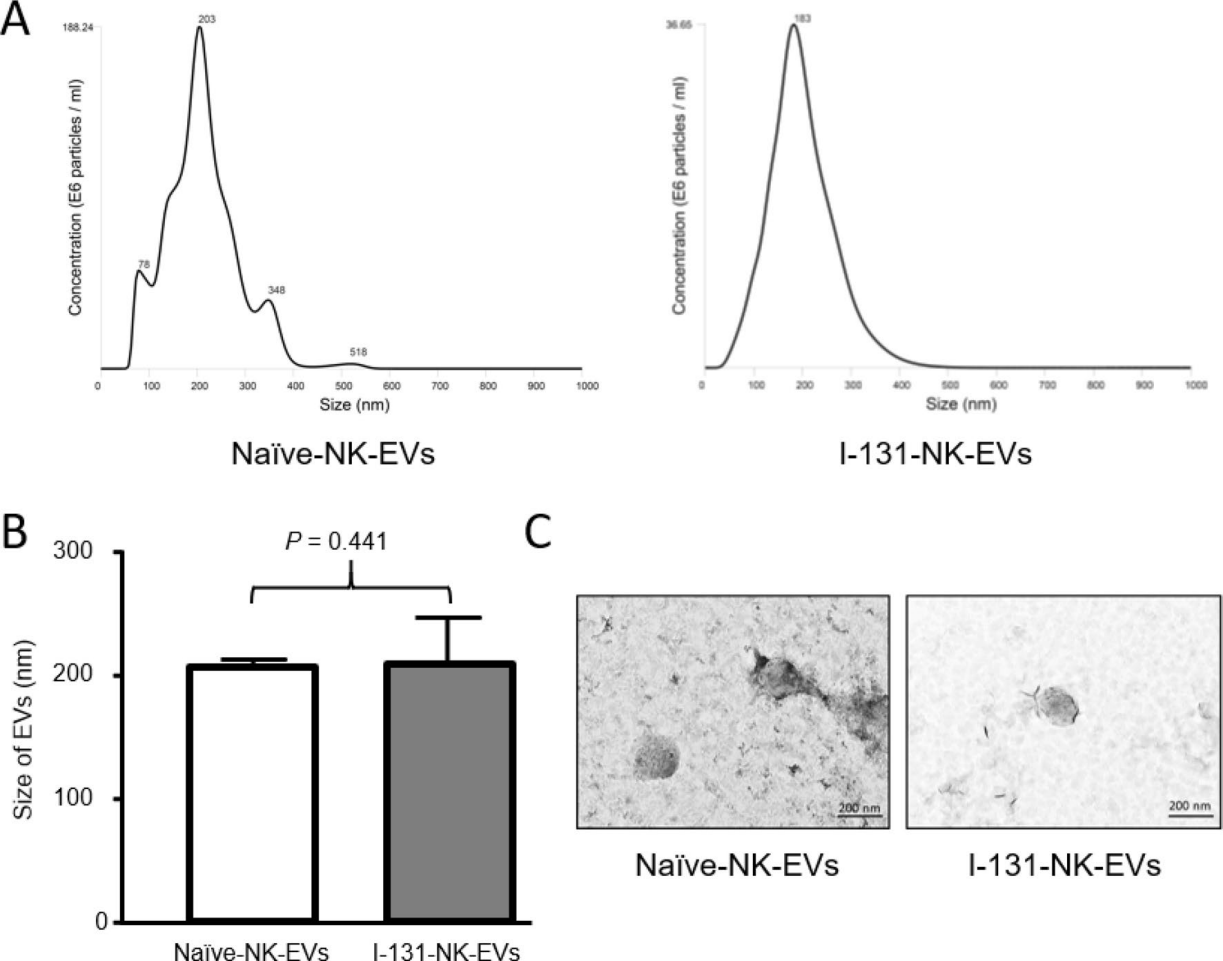
Characterization of I-131-NK-EVs. (**A**) Size distributions of naïve-NK-EVs and I-131-NK-EVs were examined by nanoparticle tracking analysis. (**B**) There was no significant difference of average sizes between naïve-NK-EVs and I-131-NK-EVs. (**C**) There were no significant differences of morphology and size between naïve-NK-EVs and I-131-NK-EVs at transmission electron microscopy images. Data are presented as mean ± standard deviation.

There were no significant differences in sizes between naïve-NK-EVs (207.7 ± 4.1 nm), I-131-NK-EVs (233.8 ± 32.7 nm) (Fig. 4A,B). Morphology and size of EVs were also similar in transmission electron microscopy (TEM) images (Fig. 4C).

### Gamma camera imaging and biodistribution of Cal62-EVs

**Figure 5:**
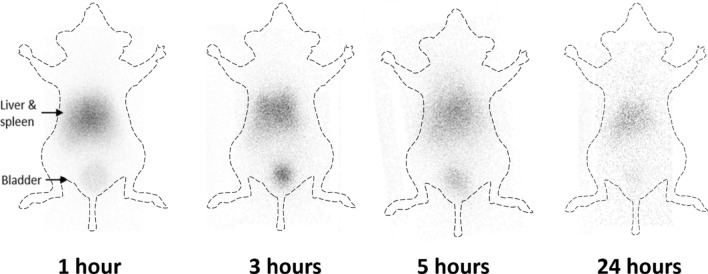
In vivo imaging of I-131-Cal62-EVs. After intravenous injection of I-131-Cal-62-EVs (3.7 GBq), gamma camera images were acquired at 1 h, 3 h, 5 h and 24 h in BALB/c nude mice. The gamma camera images showed intense uptake in liver and spleen area. And there shows intense trace accumulation at bladder.

In vivo imaging of I-131-Cal62-EVs was acquired in mice by gamma camera. The serial images acquired at 1 h, 3 h, 5 h and 24 h after intravenous injection showed that I-131-Cal62-EVs were taken up mainly by the liver and spleen, and thyroid gland and stomach uptakes were not visualized on all of the images (Fig. 5). The serial images showed decreased tracer uptake in the mouse, and the tracer was excreted through urine.

**Figure 6:**
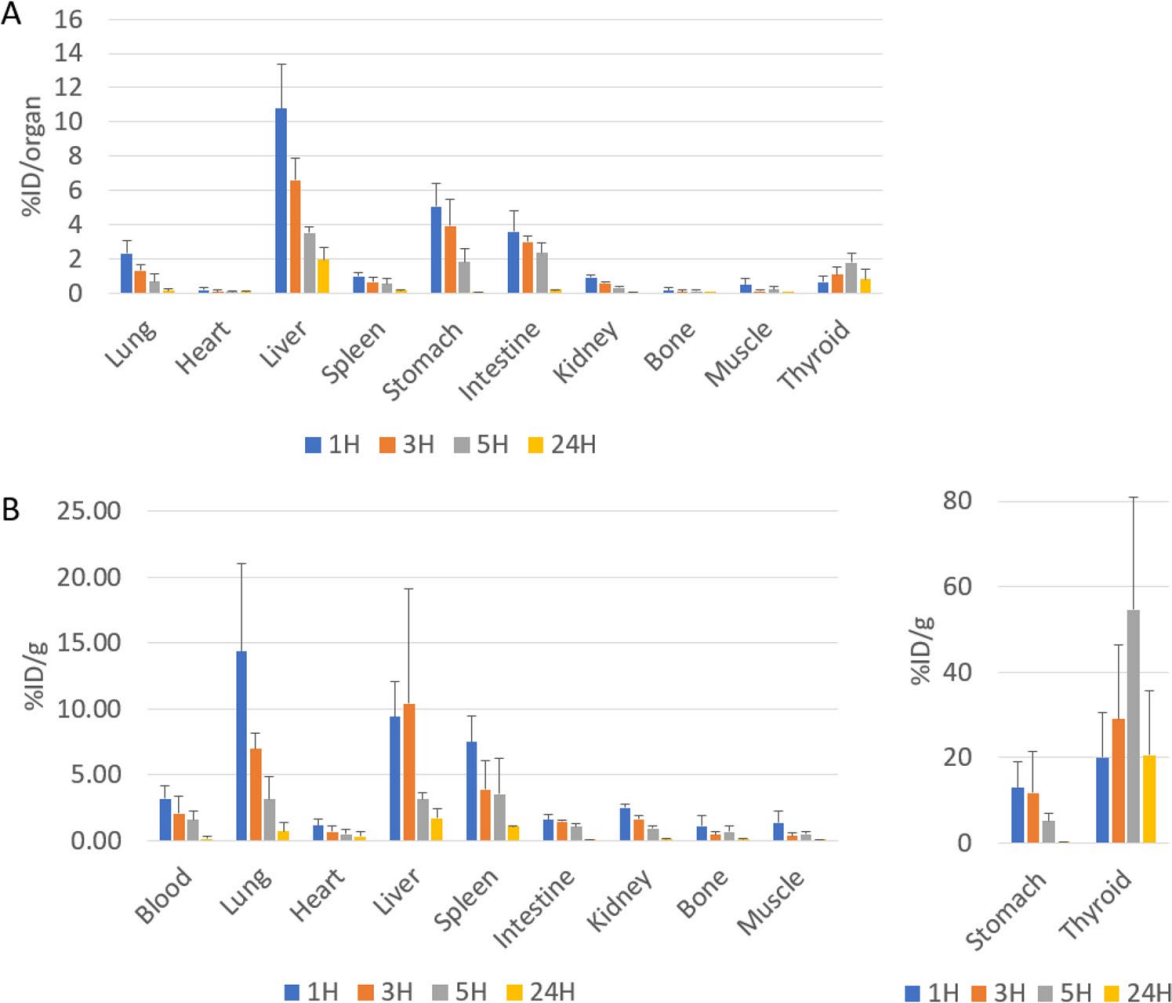
Ex vivo biodistribution of I-125-Cal62-EVs. I-125-Cal62-EVs (111 kBq) were intravenously injected to mice, and mice were sacrificed at 1 h, 3 h, 5 h (n = 3 for each group) and 24 h (n = 4) after injection. (**A**) %ID/organ represents counts of radioactivity normalized by injected dose per organ. (**B**) %ID/g represents counts of radioactivity normalized by injected dose per gram of organ. Data are presented as mean ± standard deviation.

For biodistribution study, mice were sacrificed after 1 h, 3 h, 5 h, and 24 h after intravenous injection of I-125 labeled Cal62 EVs (I-125-Cal62-EVs). I-125-Cal62-EVs are mainly taken up by lung, liver and spleen. I-125-Cal62-EVs in lung, heart, liver, spleen and kidneys were decreased in serial time points (1 h, 3 h, 5 h and 24 h). Liver showed highest uptake, and % uptakes of injected dose (%ID) for liver were 10.8 ± 2.6 at 1 h, 6.6 ± 1.3 at 3 h, 3.5 ± 0.4 at 5 h and 1.9 ± 0.7 at 24 h, respectively. While, those of lung were 2.3 ± 0.7 at 1 h, 1.3 ± 0.4 at 3 h, 0.7 ± 0.4 at 5 h and 0.1 ± 0.1 at 24 h. And those of spleen were 1.0 ± 0.2 at 1 h, 0.6 ± 0.3 at 3 h, 0.5 ± 0.3 at 5 h and 0.2 ± 0.0 at 24 h, respectively (Fig. 6A). And %ID/g was also calculated and demonstrated in Fig. 6B.

## Discussion

Recent studies have presented that EV labeled with In-111-oxine or Tc-99m were successfully used for in vivo trafficking EVs^16, 17, 19^. Smyth et al. labeled EVs with In-111-oxine and analyzed their biodistributions^19^. But they did not perform gamma camera or SPECT imaging. In addition, In-111 released from EVs might be accumulated in the reticuloendothelial system, that signals cannot be discriminated between free In-111 or In-111 labeled EVs. In-111 is preferable for delayed imaging due to its long half-life (2.8 days). Because of relatively short half-life of Tc-99 m (6 h), it is not appropriate for delayed image. But Tc-99m is much cheaper and provides better image quality than In-111. Therefore, several previous studies have tried to label Tc-99 m to EVs.

Hwang et al. labeled EV mimetics with Tc-99m hexa-methyl-propylene -amine-oxime (HMPAO), and imaged the EV mimetics successfully using SPECT^17^. Tc-99m-HMPAO, a lipophilic material, penetrates the membrane of EV mimetics and reacts with glutathione. As glutathione concentration of the EV might affect the radiochemical yield of the EV. EV from different cells have different concentrations of glutathione, radiochemical yield of EV using Tc-99m HMPAO might be variable depends on parent cells of the EVs^2, 20^. Tc-99m-tricarbonly was also used for labeling EVs, and it provides relatively higher radiochemical yield (38.8%)^21^. However, it only provided 1 h image after injection, that further investigations might be needed to verify its efficacy for delayed in vivo imaging of EVs. Red blood cell (RBC) derived EVs and EV mimetics contain hemoglobin that Tc-99m can be simply labeled to RBC derived EVs and EV mimetics using tin (II) chloride (18). This labeling method provides excellent radiochemical yield (> 95%) and good serum stability. However, this method only can be used for RBC derived EVs and EV mimetics.

Iodination is widely used for labeling radioiodine to peptides, antibodies, proteins and cells for a long time^22^. Radioiodines have various species of radioisotopes, including I-123, I-124, I-125 and I-131. They have different physical properties, such as types of emitting radiation and half-life. I-123 and I-131 (half-life: 13.2 h and 8.0 days) are widely used for gamma camera and SPECT imaging, while I-124 (half-life: 4.2 days) is used for PET imaging. I-125 is widely used for biodistribution study and laboratory tests, due to its long half-life (59.5 days) and low energy level of emitting radiation^11^. These radioisotopes have same chemical properties, due to same proton number, EVs can be labeled with different kinds of radioiodines using the same method.

Recently, Rashid et al. showed I-131 labeling of EVs and in vivo imaging using SPECT^23^. They labeled I-131 to tyrosine of membrane proteins of EVs using iodination beads. However, the images show high uptake of thyroid gland and they did not show radiochemical yield of the method. In the current study, gamma camera images revealed no visible thyroid uptake. To increase the iodination sites of membrane protein, sulfo-SHPP was labeled to the membrane proteins of EVs and then iodination to SHPP was performed. Therefore, this method showed good radiochemical yield and excellent radiochemical purity (> 98%) after purification.

Even though, serum stability test showed that about 90% of radioiodine were still labeled to the EV at 24 h, gamma camera images showed bladder uptake. We assume that this result might be associated with the decomposition of EVs. Thyroid uptake in ex vivo biodistribution data and bladder uptake of the image might be associated with the free I-131. The previous studies using radioiodination of EVs were also showed urinary excretion and thyroid uptake of free radioiodine^18, 23^. The thyroid uptake of radioiodine was not high enough to be visualized in the gamma camera images, but ex vivo biodistribution study can sensitively detect the radioiodine accumulation. As the thyroid is small organ, %ID/g can be calculated as relatively high.

In the current study, tumor cell derived EVs and NK cell derived EVs were successfully labeled with radioiodine. Before and after radioiodine labeling, size and morphology of EVs were not significantly different. The radioiodine labeling method can be applied to any kind of EVs regardless of characteristics of origin cells.

Serial gamma camera images showed persistent EV uptakes in liver and spleen, and these findings were consistent with data of ex vivo biodistribution analysis. Although fluorescence and bioluminescence images had been used to in vivo tracking of EV and the images also showed liver and spleen uptake^8, 10^, but signal character of the optical imaging was not good enough to evaluate quantitatively due to limited tissue penetration power of them. However, when nuclear imaging is applied to assess in vivo EV distribution, excellent penetration power of the gamma ray provides accurate quantitative data about EV biodistribution^5, 6, 24, 25^. In addition to innate therapeutic ability of EVs, drugs, proteins and microRNA can be loaded to EVs and deliver them to target lesions. Accurate trafficking of these EV is helpful for predicting efficacy of the EVs as the drug carrier. This radioiodine imaging technique can be applied for these studies in the future.

There are several limitations in the current study. As sulfo-SHPP was labeled to membrane proteins of EVs, there might be modifications of the proteins. Although gamma camera imaging did not visualize the thyroid glands, there was free radioiodine which released from the radiolabeled EV in ex vivo biodistribution. The free radioiodine cannot be discriminated from EV labeled radioiodine. Pin-hole gamma camera imaging has limited resolution, that quantification measurements based on the images cannot be performed at the current study. Recent advanced SPECT or PET provide higher resolutions and organ segmentations, that quantification measurements based on in vivo images could be available in future study.

Nuclear imaging system of EVs derived from thyroid cancer and NK cells using radioiodine labeling of the EVs was successfully established. And this in vivo tracking system of EVs can be helpful for development EV theranostics.

## Materials and methods

### Cell culture

An anaplastic thyroid cancer cell line (ATC), Cal-62, and the human NK cell line, NK92-MI, which was obtained from the ATCC. Cal-62 cells were cultured in Dulbecco’s modified Eagle medium (DMEM, Gibco, Grand Island, NY, USA) supplemented with 10% fetal calf serum (Hyclone, Logan, UT, USA) and 1 antibiotics (Gibco, Grand Island, NY, USA). NK92-MI cells were cultured in stem cell growth medium (Cellgro, Freiburg, Germany) supplemented with 2% human serum and 1% antibiotics. These cells were incubated at 37 °C in a 5% CO_2_ atmosphere.

### Isolation of EVs

For isolation of EVs, EV-depleted serum was prepared. Serum was filtered through a 0.22 µm syringe filter and then centrifuged for 18 h at 100,000 × g at 4 °C. EV-depleted serum was used for culturing the cells for 3 days. We isolated the EVs from the culture medium as described previously^8, 24^. Briefly, the supernatant of media was sequentially centrifuged at 300 × g for 10 min, 1500 × g for 20 min, and finally 2500 × g for 20 min. Then, the supernatant was filtered through a 0.45 µm syringe filter and centrifuged at 100,000 × g for 60 min. The pellet was resuspended with PBS and centrifuged at 100,000 × g for 60 min. The final pellet was resuspended in 50–100 µL of PBS and stored at − 80 °C. Isolated EVs were used within one week. All ultracentrifugation steps were performed using SW-28 rotor, the OptimaTM L-100 XP ultracentrifuge (Beckman Coulter, Brea, CA, USA). All centrifugation steps were conducted at 4 °C.

### Radionuclide labeling of EVs

Immediately before use, 5 mg of sulfosuccinimidyl-3-(4-hydroxypheynyl) propionate (sulfo-SHPP, ThermoFisher, Rockford, IL, USA) was dissolved in 1 mL of PBS. 100 µL of sulfo-SHPP were added to 100 µL of EVs (1 mg/mL) and incubated in 4° C for 3 h with gentle mixing. The sample was centrifuged at 100,000 × g in 4° C for 60 min and the pellet was resuspended in 100 µL of PBS (SHPP-EVs). Pierce Pre-Coated Iodination Tubes, formerly called “IODO-GEN”, were used for radioiodination. Pre-coated iodination tube was wet with PBS and 10 µL (37 MBq) of I-131 or I-125 was added to the tube. The tube was gently mixed for 5 min at room temperature to activate radioiodine. Then, prepared SHPP labeled EVs were added to the tube and incubated for 15 min at room temperature with gentle mixing. The sample was centrifuged at 100,000 × g in 4 °C for 60 min and the pellet was resuspended in 100 µL of PBS. Radiochemical purity was measured by instant thin-layer chromatography (ITLC) using 0.9% NaCl solution as an eluent for each column. Radioactivity of the column was counted using a TLC imaging scanner (AR-2000, Bioscan, Poway, CA, USA). Radiochemical yield was calculated as radioactivity of radioiodine labeled EVs after purification divided by radioactivity of pre-labeled radioiodine (n = 3).

### Electron microscopy

We prepared samples following previous protocol^8^. Briefly, pellets of EVs were resuspended in 100 µL of 2% paraformaldehyde. Then, 5 µL of EVs and I-131-EVs were individually attached to the Formvar-carbon coated with EM grids (Electron Microscopy Sciences, USA) and dried and washed. The grids were then placed on 1% of glutaraldehyde and incubated at room temperature and then washed with distilled water. The EVs in the grids were stained with 2% uranyl acetate. Then, the grids were washed seven times with PBS, allowed to completely dry. The samples were observed on Titan G2 ChemiSTEM with a Cs Probe (FEI company, Netherlands) to measure the size of the EVs. Scanning electron microscopy (SEM) and transmission electron microscopy (TEM) images were obtained.

### Nanoparticle tracking analysis

The sizes of EVs were measured using Nano Sight LM 10 (Malvern Instruments Limited, Worcester, UK). The measurements were performed according to the provided instruction as described previously^8^. The measurements were done in triplicate and analyzed.

### Stability test

I-131 labeled Cal62-EVs were incubated in 20% fetal bovine serum for 1 h, 2 h, 4 h and 24 h after isolation. The serial serum stability was calculated by ITLC using 0.9% NaCl solution as eluent. Stability was determined by the percent change in the radiochemical purity of I-131 labeled Cal62-EVs over time. The same procedures were done in triplicate and analyzed.

### Animals

Five-week-old female BALB/c nude mice were purchased from Japan SLC, Inc. (Shizuoka, Japan). All procedures were reviewed and approved by Review Board of Kyungpook National University. All methods were carried out in compliance with ARRIVE guidelines and regulations of the local animal welfare ethics committee. The mice were anesthetized with 2% isoflurane (Merial, Lyon, France) and I-131-labeled Cal62-EVs (3.7 MBq) were intravenously injected into the mice (n = 3). Images were captured at 1 h, 3 h, 5 h and 24 h using a pin-hole gamma camera for 10 min (Infinia, GE, Milwaukee, WI, USA).

For serial biodistribution study, Cal62-EVs were labeled with I-125 (I-125-Cal62-EVs). Mice were sacrificed at 1 h (n = 3), 3 h (n = 3), 5 h (n = 3) and 24 h (n = 4) after intravenous injection of I-125-Cal62-EVs (111 kBq), and major organs were dissected for measurement. The radioactivity of each organ was measured with a gamma counter (Cobra, Packard, Ramsey, MN, USA).

### Statistical analysis

All data are expressed as the means ± standard deviation (SD). Two groups of data were statistically analyzed by t-test. We analyzed data by using R version 3.5.3 software (https://www.r-project.org)^26^, and a *p* value less than 0.05 was considered statistically significant.
